# Sight or Scent: Lemur Sensory Reliance in Detecting Food Quality Varies with Feeding Ecology

**DOI:** 10.1371/journal.pone.0041558

**Published:** 2012-08-03

**Authors:** Julie Rushmore, Sara D. Leonhardt, Christine M. Drea

**Affiliations:** 1 Department of Evolutionary Anthropology, Duke University, Durham, North Carolina, United States of America; 2 Odum School of Ecology, University of Georgia, Athens, Georgia, United States of America; 3 Department of Ecology, Leuphana University, Lüneburg, Germany; 4 Department of Biology, Duke University, Durham, North Carolina, United States of America; University of Sussex, United Kingdom

## Abstract

Visual and olfactory cues provide important information to foragers, yet we know little about species differences in sensory reliance during food selection. In a series of experimental foraging studies, we examined the relative reliance on vision versus olfaction in three diurnal, primate species with diverse feeding ecologies, including folivorous Coquerel's sifakas (*Propithecus coquereli*), frugivorous ruffed lemurs (*Varecia variegata* spp), and generalist ring-tailed lemurs (*Lemur catta*). We used animals with known color-vision status and foods for which different maturation stages (and hence quality) produce distinct visual and olfactory cues (the latter determined chemically). We first showed that lemurs preferentially selected high-quality foods over low-quality foods when visual and olfactory cues were simultaneously available for both food types. Next, using a novel apparatus in a series of discrimination trials, we either manipulated food quality (while holding sensory cues constant) or manipulated sensory cues (while holding food quality constant). Among our study subjects that showed relatively strong preferences for high-quality foods, folivores required both sensory cues combined to reliably identify their preferred foods, whereas generalists could identify their preferred foods using either cue alone, and frugivores could identify their preferred foods using olfactory, but not visual, cues alone. Moreover, when only high-quality foods were available, folivores and generalists used visual rather than olfactory cues to select food, whereas frugivores used both cue types equally. Lastly, individuals in all three of the study species predominantly relied on sight when choosing between low-quality foods, but species differed in the strength of their sensory biases. Our results generally emphasize visual over olfactory reliance in foraging lemurs, but we suggest that the relative sensory reliance of animals may vary with their feeding ecology.

## Introduction

As foraging is a costly behavior, animals enhance their efficiency for detecting differences in food quality via sensory adaptations [1]. Animals can also adjust their food selection to regulate nutrient intake [2]–[4] or to avoid plant secondary metabolites that may be harmful or difficult to digest (reviewed in [5]). Two main senses involved in food detection and selection by mammals are sight [6]–[8] and scent [9], [10]. Whereas the use of these senses during foraging is well recognized, further work is needed to elucidate the relative contribution of vision versus olfaction in enhancing the food-quality choices of multisensory animals. In a comparative study of Malagasy lemurs (Primates; Strepsirrhini), we use an experimental approach to investigate species differences in sensory reliance. We compare three diurnal species that have different feeding ecologies, including frugivory and folivory, as we expect that dietary specializations may require differential emphasis on these two senses.

Strepsirrhine primates (lemurs, lorises, and galagos) represent a particularly interesting group in which to investigate the importance of visual and olfactory senses in foraging behavior, as their sensory abilities appear to be intermediary between those of other mammals and those of haplorhine primates (tarsiers, monkeys, and apes). Notably, relative to other mammals, primate evolution is marked by a general decrease in olfactory sensitivity and reliance (reviewed in [11], [12], but see [13]) and an increase in visual abilities, such as enhanced depth perception [14], [15]. Within these broad characterizations, strepsirrhines have a better-developed sense of smell than do haplorhines, including a functional vomeronasal organ [16], but have decreased visual acuity [17] and are more routinely dichromatic [18]. As trichromatic color vision in foraging monkeys and apes purportedly facilitates the detection of ripe fruits [19]–[21] or young leaves [22]–[24], lemurs might be expected to rely more heavily on their olfactory sense for food detection (see Text S1 for further discussion of strepsirrhine trichromacy).

In a recent study of 50 mammalian genomes, researchers showed that ecotype better predicted olfactory receptor (OR) gene repertoires than did phylogenetic relatedness, suggesting strong ecological adaptation within at least one sensory modality [25]. We further suspect that the relative reliance on visual versus olfactory cues during foraging might vary by an animal's dietary specialization, reflecting the availability, distribution, or cue salience of different foods. Here, we address cue salience from the perspective of the forager. From the perspective of the plant, however, fruits should be more conspicuous to consumers than should leaves, as by evolutionary design, fruit should be consumed, whereas leaves should not [26]. Accordingly, folivores that exploit broadly distributed, yet chemically subtle, leaves might rely primarily on visual cues while foraging. Frugivores that exploit more patchy distributions of highly odoriferous fruits might rely relatively more on olfactory cues. Lastly, generalists that require the flexibility to exploit a broader range of foods might depend more equally on both senses, but may sacrifice some aspect of their sensory specialization or efficiency to do so.

Within primates, there is evidence of folivores exploiting broadly distributed leaves (e.g., [27], [28], but see [29]), as well as evidence of frugivores exploiting more patchily distributed fruits [30]. Furthermore, Dudley [31], [32] proposed that seed dispersers, such as frugivorous primates, might be attracted to low ethanol concentrations in fruits and, in a series of experimental studies, Laska and colleagues [33]–[35] have shown that several primate species are highly sensitive to fruit-related odors. To test our predictions, we selected three primarily diurnal species with different feeding ecologies, including the folivorous Coquerel's sifaka, *Propithecus coquereli* [36], [37], the frugivorous ruffed lemur, *Varecia variegata* spp. [38]–[40], and the generalist ring-tailed lemur, *Lemur catta*, that eats both fruits and leaves [41] (Table 1).

**Table 1:** Diet, sample size, and predicted sensory reliance for three species of strepsirrhine primates.

Species	Sifaka	Ruffed lemur	Ring-tailed lemur
Diet	Folivory	Frugivory	Generalist
Subjects (by sex)*	15 (7 F, 8 M)	11 (8 F, 3 M)	6 (4 F, 2 M)
Predicted sensory reliance	Visual	Olfactory	Visual & olfactory

* F, female; M, male.

Because sensory acuity is involved in food detection and evaluation, we examined lemur sensory reliance during selection of fruits and leaves at different maturational stages and, hence, representing different quality. Relative to unripe fruits, ripe fruits tend to be more caloric, contain more sugar, and have lower ratios of indigestible fibers [42], [43]. Relative to mature leaves, young leaves typically have a higher protein-to-fiber content and are richer in free amino acids [23], [44]. Vertebrates generally show a preference for ripe over unripe fruits (e.g., [45]) and for young over mature leaves (e.g., [46], [47]). The question remains as to how animals inform their decisions when selecting high-quality foods: Does their dietary specialization influence their relative reliance on visual versus olfactory properties of the food?

As fruits ripen or leaves mature, their changing nutritional quality is often accompanied by changes in visual or olfactory cues that could guide the foraging decisions of animals [42], [43], [48]. For instance, as a strawberry ripens, changing from green to red, the total amount of volatile chemicals rapidly increases, producing a stronger fruit aroma [49]. Likewise, the volatiles of ripe, red tomatoes are associated with essential nutrients, including fatty acids, amino acids, and carotenoids [43]. Primates readily distinguish differences in color or brightness (e.g., in trichromatic species [50], e.g., in dichromatic species [51], see also [52]). Moreover, given the qualitative and quantitative differences between the volatile compounds emitted by ripe and unripe fruits [53], [54] or between young and mature leaves [55], [56], animals might be able to discriminate the maturational state or nutritional value of foods by olfactory cues alone.

Using food items for which we confirmed the presence of multimodal signals (in that maturation is reflected by changes both in visual and olfactory cues), we first examined if lemurs select the more nutritious over less nutritious food when both sensory cues and both food types are simultaneously available. When given a choice between young leaves and mature leaves, sifakas should prefer the more nutritious, young leaves. Likewise, ruffed lemurs and ring-tailed lemurs should prefer the more nutritious, ripe fruits over unripe fruits.

We then used an experimental approach to tease apart the variables contributing to these food choices. Using a novel discrimination apparatus, we manipulated the sensory cues and developmental states (i.e., quality) of the foods available to the lemurs. We tested if the lemurs could detect qualitative differences in the various food items using either visual or olfactory cues alone. We also examined if the lemurs relied more on one sensory modality over the other when selecting between comparable food items. Our research questions explore whether species-specific sensory reliance relates to different feeding ecologies.

## Materials and Methods

### Subjects and Housing

Our subjects were 32 strepsirrhine primates from the Duke Lemur Center (DLC) in Durham, NC (Table 1, Table S1). The ruffed lemurs included two subspecies, red-ruffed lemurs (*V. v. rubra*) and black-and-white ruffed lemurs (*V. v. variegata*), which we treated as a single group because of their close genetic relatedness and similar feeding ecologies [38], [39]. All of the subjects were subadult or adult at the time of study, ranging in age from 1–30 years.

In keeping with their different diets, the sifakas were provisioned primarily with leaves (e.g. mimosa, sweet gum, sumac, tulip poplar) and Leaf-Eater Primate Diet mini-biscuits (Mazuri, St. Louis, MO), but also received vegetables, including leafy greens (e.g. collards, kale), nuts or beans and, occasionally, fruit. The ruffed lemurs received primarily fruit and monkey chow (Monkey Diet^TM^, PMI Feeds, St. Louis, MO), but also vegetables. The ring-tailed lemurs received primarily monkey chow, and equal proportions of fruits and vegetables. Although seasonally variable, the fruit (e.g. apple, banana, grape, kiwi, mango, melon, peach, pear, plum) and vegetable (e.g. beet, broccoli, cabbage, carrot, cauliflower, corn, cucumber, green beans, greens, squash, sweet potato) options were comparable across species. Water was freely available.

The animals were housed socially in small groups of 2–6 animals. Testing occurred from December 2005–July 2006 in the animals' ‘indoor’ enclosures (5–15 m^2^ at the base, 5 m in height), which had suitable enrichment, natural light, and were enclosed by chain-link fencing that allowed exposure to the elements. To encourage interest in the foraging task during experimental trials, we delayed morning feeding until after testing (at around 11:00 h). The animals were maintained in accordance with United States Department of Agriculture regulations and with the National Institutes of Health Guide for the Care and Use of Laboratory Animals. Protocols were approved by the Institutional Animal Care and Use Committee of Duke University (protocol #A314-05-10).

### Subject Visual Status

Opsin gene polymorphism that produces trichromacy in heterozygous females occurs in the Coquerel's sifaka and both subspecies of ruffed lemur, but not in ring-tailed lemurs [51], [57], [58]. We investigated individual visual status via genotyping [51] to test for a potential influence of opsin gene polymorphism (i.e., dichromacy versus trichromacy) on the performance of our subjects (Text S1). Because we found no consistent difference in trial performance by visual status, we collapsed dichromats and trichromats for the remaining analyses.

### Test Food Items

While accommodating the feeding specializations and food preferences of the different species under study, we selected test foods that were relatively novel to the animals (i.e., foods that were not in the subjects' daily diets and were rarely provided) and that occurred in both red and green maturational stages. We tested the folivores with young, red leaves versus mature, green leaves of *Photinia spp.,* a plant species native to Japan, but frequently found in North Carolina. We selected this plant because it was readily abundant, was approved by the DLC for consumption by lemurs, and its red and green maturational stages were accompanied by different chemical profiles (see results). We tested the frugivores and generalists with unripe, green versus ripened, red tomatoes (*Solanum lycopersicum*) or strawberries (*Fragaria spp.*). We used strawberries for a small subset of lemurs (*n* = 2) that, otherwise, were not motivated to participate in the experimental trials. Although color signaled different maturational stages for leaves and fruits, respectively, we assumed that the red stage was consistently the most nutritious, as young leaves and ripe fruits tend to be more nutritious than mature leaves and unripe fruits, respectively [23], [42]–[44]. Hereafter, we refer to the higher-quality, young leaves and ripe fruits as ‘red foods’ and to the lower-quality, mature leaves and unripe fruits as ‘green foods.’ We predicted that all lemurs should prefer the higher-quality, red foods.

### Extraction and Characterization of the Volatile Compounds in Test Food Items

To confirm that both red and green stages of the food items had the potential to be differentiated based on their olfactory profiles, we analyzed differences in the volatile composition between young and mature *Photinia* leaves and between unripe and ripe tomatoes using solid phase microextraction (SPME) followed by gas chromatography-mass spectrometry (GCMS). These procedures are detailed in Text S2. The semiochemical differences between ripe and unripe strawberries have been well documented [49].

### Experimental Apparatus and Foraging Tasks

**Figure 1:**
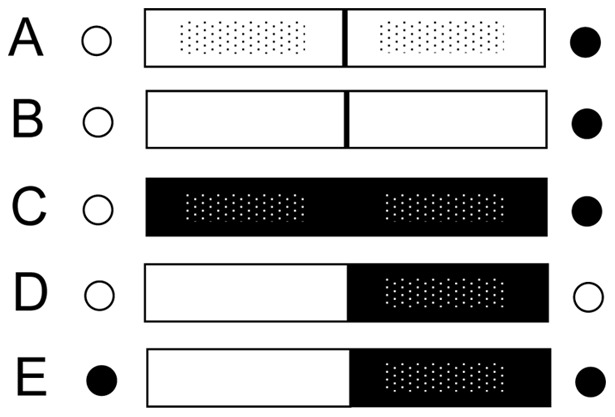
Visual representation of food choices and sensory cues presented to lemurs during experimental trials. The panels presented depict those used to test three species of strepsirrhine primates during (A) baseline, (B) visual, (C) olfactory, and (D–E) multi-sensory trials. During baseline, visual, and olfactory trials (A–C), both a red and a green food item were presented within a trial. During multi-sensory trials (D–E) food-quality was held constant within a trial. Black circles represent red food items (i.e., young leaves or ripe fruits); white circles represent green food items (i.e., mature leaves or unripe fruits). White rectangles represent clear panels that provided visual access to food items; black rectangles represent opaque panels that blocked visual access. Rectangles with dots represent pierced panels that provided olfactory access to food items; rectangles without dots represent solid panels that blocked olfactory access.

**Figure 2:**
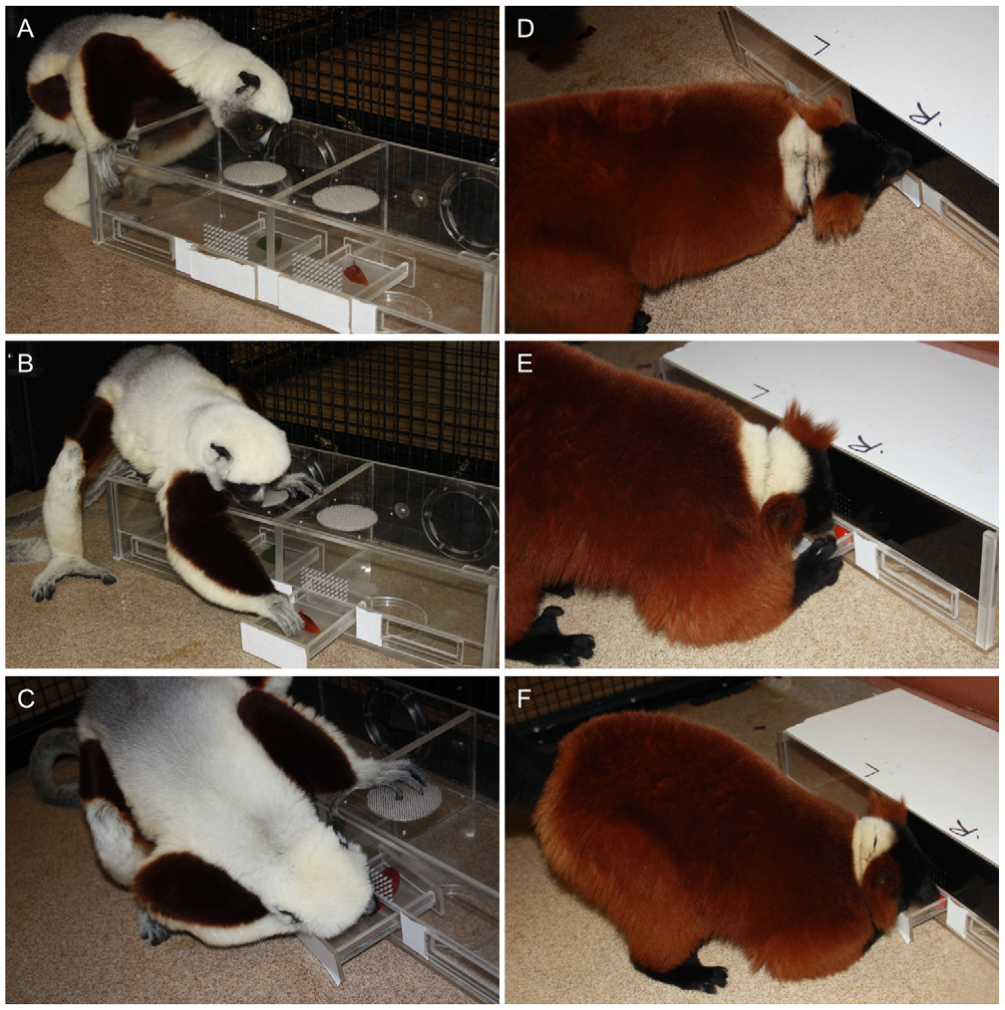
The foraging tasks. A representative (A–C) sifaka and (D–F) ruffed lemur solving different foraging tasks presented to three species of strepsirrhine primates. Shown are (A–C) a baseline trial depicting visual reliance by the sifaka and (D–F) a multisensory trial depicting olfactory reliance by the ruffed lemur. In both tasks, the animal must (A, D) investigate the box contents using whatever sensory information is available or preferred, (B, E) manually open one of the two drawers, thereby making its choice, and (C, F) orally retrieve the selected food item.

**Figure 3:**
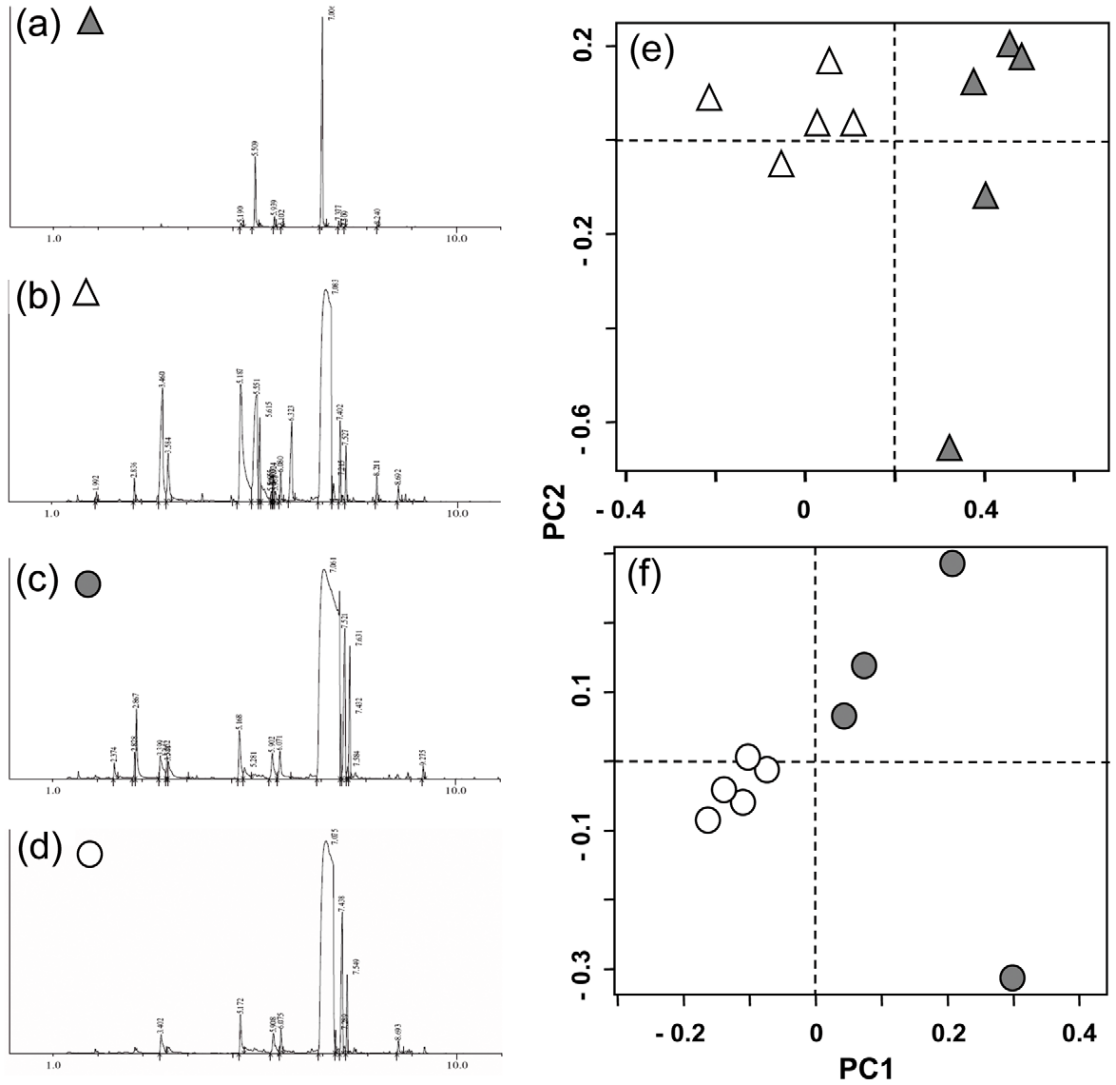
Chromatograms and principal component analysis of the chemical components in test-food items. Representative chromatograms are shown for (A) red, young leaves (*Photinia:* shaded triangle), (B) green, mature leaves (open triangle), (C) red, ripe tomatoes (*Solanum lycopersicum*: shaded circle), and (D) green, unripe tomatoes (open circle), obtained by solid phase microextraction, followed by gas chromatography and mass spectrometry. Also shown are the results from the respective principal component analyses (PCA) of the chemical components identified in both red and green (E) leaves and (F) fruit. Dashed lines represent the two main PC axes.

We used a two-choice paradigm to test the subjects' foraging decisions, using a novel apparatus that allowed manipulating the sensory cues and foods available. The apparatus was a professionally constructed Plexiglas box (50 cm×15 cm×25 cm) partitioned into two compartments of equal size. Each compartment contained a food item accessible by a small drawer (8 cm×12 cm) located beneath a ‘sensory’ panel. Depending on which of the interchangeable sensory panels we inserted across the top half of the box front, the contents of each compartment could be advertised by various combinations of visual and olfactory cues (Figure 1). Our combinations were as follows: (1) Open drawers or a pierced, transparent panel provided both visual and olfactory cues about the compartment contents (Figure 1A, Figure 2A–C); (2) a solid, transparent panel provided only visual cues about the compartment contents (Figure 1B); (3) a pierced, opaque panel blocked visual access, but allowed the emission of olfactory cues (Figure 1C); and (4) a ‘multisensory’ panel provided visual cues for one compartment, but olfactory cues for the other compartment (and could be reversed to control for side biases, Figure 1D–E, Figure 2D–F). The task for the animal facing the box was to investigate its contents using the sensory information available (Figures 2A, D), manually open one of the two drawers (Figures 2B, E), and retrieve a food item, usually orally (Figures 2C, F).

### Apparatus Validation with Electronic Sensor Technology

Given the depth of the Plexiglas®, the piercings in our apparatus were too small to allow visual access via the opaque panels. Likewise, the sensory panels slid tightly into place to keep the box as airtight as possible, thereby preventing olfactory access via the solid panels. Nevertheless, we used an electronic sensor (zNose®, Model 4300, Electronic Sensor Technology, Newbury Park, CA) to verify that olfactory cues were available from the pierced panels, but not from the solid panels (see Text S3 for details on zNose® technology and on our methodology).

The results of our validation procedures confirmed that the solid and pierced panels served their respective functions to block or allow the passage of food-related olfactory cues ( Figure S1). More specifically, the muted chromatograms derived from an empty, open drawer (Figure S1A), equivalent to control room air, were comparable to those derived from a food item behind a solid panel (Figure S1B), suggesting that food-related odor cues were unavailable to lemurs under the ‘visual’ condition. By contrast, the pronounced peaks displayed in the chromatograms derived from the same food item behind a pierced panel suggested that food-related olfactory cues were available to the lemurs under the ‘olfactory’ condition (Figure S1C).

### Experimental Testing Procedures

#### Habituation to the foraging apparatus and task for estimating baseline preferences

Over a period of five days, we habituated the subjects to the apparatus for 30–60 min per day by allowing them to interact with the box and open the drawers to receive a non-test food item. The subjects continued habituation until they had demonstrated the ability to open a drawer to retrieve a food item and a propensity to sniff or look at both drawers before opening one of them (Figure 2). After meeting these criteria, the subjects began baseline trials during which we simultaneously presented them with red and green food items, to establish individual preferences. We began by holding the food items 30 cm from the animal's nose (initially allowing the subjects to look at, sniff, or lick each food item prior to choosing one). Then, we gradually presented the food items using the foraging apparatus, either with opened drawers or with the ‘baseline’ panel, which provided visual and olfactory cues. During these and all subsequent experimental trials, we randomly varied the side on which we presented the red food item to prevent side biases.

#### Experimental test sessions for evaluating sensory reliance

All of the experimental test sessions incorporated the foraging apparatus (Figure 1) and typically spanned two days. Each test day began with 10 consecutive baseline trials after which each subject participated in a subset of 8 randomly presented experimental trials, including visual (*n* = 2), olfactory (*n* = 2), and multi-sensory (*n* = 4) trials. To counterbalance the position of the cues and control for any potential side bias, we presented each possible choice simulation twice (totaling *n* = 20 baseline and *n* = 16 experimental trials) per subject. We cleaned the drawers with water after each trial. Test sessions occurred during weekdays, between 0800–1200 h and produced a total of 70 h of behavioral tests. We used a handheld Psion computer and the Observer software (Noldus Information Technology, Leesburg, VA) for data collection. For all trials, we recorded the food item selected by each subject. As an additional means to test relative reliance on visual versus olfactory cues, and to assess if generalist ring-tailed lemurs might sacrifice efficiency (consistent with the speed-accuracy trade-off observed in other species [59]), we scored the duration with which the ruffed lemurs and ring-tailed lemurs looked at and sniffed the apparatus prior to making a food selection. The methods for these latter tests are presented in Text S4.

### Statistical Analyses

#### Food preferences and sensory reliance of lemurs during baseline and experimental trials

To investigate baseline preferences for red versus green foods, we analyzed the frequency of food choices by species and by individual, using goodness-of-fit *G*-tests [60]. We designed the subsequent visual and olfactory trials specifically to test the sensory reliance of animals selecting their *preferred* food. Therefore, we could only include in the analyses of those later trials the animals that showed a preference during baseline trials. Because retaining only those animals that showed a statistically significant preference (i.e., at *P*<0.05 by *G*-test) would have restricted the sample sizes, we additionally identified those subjects that tended to show individual preferences (i.e., at *P*<0.10 by *G*-test) during the baseline trials and retained those combined subjects (i.e., showing relatively strong preferences at *P*<0.05 or *P*<0.10) in subsequent *G-*tests involving visual and olfactory trials. Nevertheless, to evaluate any potential effect of our selection criterion, we provide comparable results derived from retaining (a) all of the subjects in these analyses or (b) only those showing statistically significant preferences at *P*<0.05 (see Text S5).

Because the multi-sensory trials did not involve a choice between foods of different quality (and, hence, individual food preferences were irrelevant), we reverted to using all of the subjects in these analyses. To investigate which sensory cue the different species relied on most during these trials, we determined the number of times each animal used visual versus olfactory cues to select both red and green foods, and then analyzed these frequencies using *G*-tests. We also used *G*-tests to examine differences among all three species in the frequency with which subjects relied on visual versus olfactory cues during multi-sensory trials.

## Results

### Chemical Differences Between Red and Green Food Items

To confirm the multimodality of our test food items, we examined their chemical profiles and then compared the profiles of red and green food items using principal component analyses (PCA, described in Text S2). We found 7 different volatile compounds associated with red, immature *Photinia* leaves (Figure 3A), compared to 13–15 compounds associated with green, mature leaves (Figure 3B). Thus, the number of volatiles emitted doubled during leaf development, such that red leaves were associated with relatively few volatiles. In *S. lycopersicum*, we detected 14–15 compounds expressed by red, ripe fruit (Figure 3C), compared to 8 volatiles expressed by green, unripe fruit (Figure 3D). Thus, the number of volatiles emitted also doubled during fruit development, but in this case, red fruits were associated with the greater number of volatiles. For both types of foods, the PCA discriminated red and green items (leaves: Figure 3E; fruit: Figure 3F). Thus, provided that the lemurs were sensitive to the items' volatile compounds, red and green *Photinia* and *S. lycopersicum* should have been distinguishable based on olfactory cues alone.

### Preference for the Nutritional Quality of Foods during Baseline Trials

**Figure 4:**
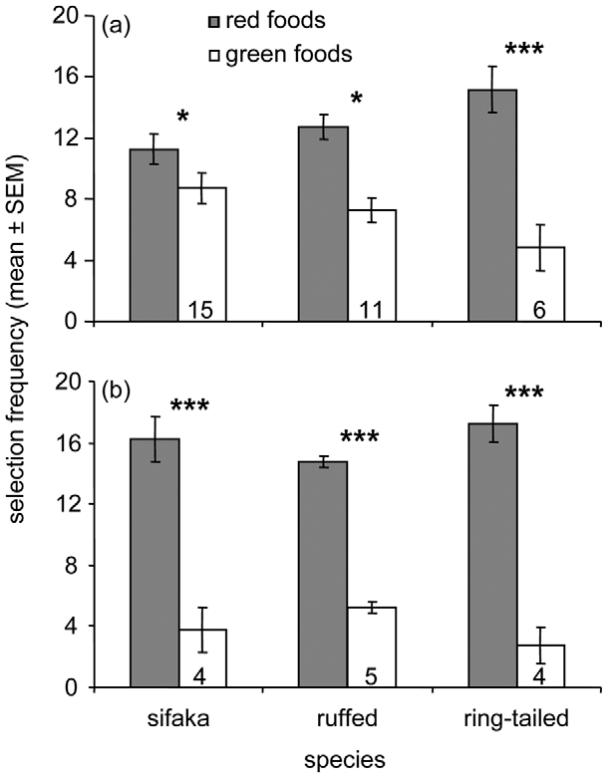
Baseline food preferences. Food choices of three strepsirrhine species are depicted for (A) all subjects tested and (B) those subjects that showed relatively strong preferences during baseline trials. Presented are the frequencies (mean ± standard error of mean) with which the animals selected red foods (i.e., young leaves or ripe fruits; shaded bars) versus green foods (i.e., mature leaves or unripe fruit; open bars) when both visual and olfactory cues were simultaneously available. Numbers at the bottom of the open bars represent the number of individuals used in the analyses (G-test: * *P*<0.05, *** *P*<0.001).

During the 20 baseline trials, all three species readily solved the task (Figure 2A–C, Video S1) and showed the predicted preference for red foods (Figure 4A). Sifakas preferred red over green leaves (*n* = 15, pooled *G*_1_ = 4.83, *P*<0.05); likewise, ruffed lemurs (*n* = 11, pooled *G*_1_ = 16.57, *P*<0.001) and ring-tailed lemurs (*n* = 6, pooled *G*_1_ = 33.64, *P*<0.001) preferred red over green fruits. For all three species, the *G*-tests revealed strong heterogeneity scores (sifakas: *G*_14_ = 50.88, *P*<0.001; ruffed lemurs: *G*_10_ = 16.34, *P* = 0.09; ring-tailed lemurs: *G*_5_ = 20.44, *P*<0.01), indicating that, within species, individuals differed in the intensity of their preferences. Individually, however, no animal showed a significant preference for the green foods, whereas two sifakas, three ruffed lemurs, and three ring-tailed lemurs showed significant preferences for the red foods (*G*_1_>5.23, *P*<0.05). An additional two sifakas, two ruffed lemurs, and one ring-tailed lemur showed trends in the same direction (*G*_1_ = 3.29, *P*<0.10). We combined the individuals showing significant preferences (*P*<0.05) with those showing trends for preferentially selecting red food items (*P*<0.10) into a subset we refer to as showing ‘relatively strong’ preferences (Figure 4B). To test the sensory reliance of animals preferentially selecting high-quality foods, we retained in the subsequent analysis of visual and olfactory trials only those subjects that showed relatively strong, red-food preferences during the baseline trials.

### Preference for the Nutritional Quality of Foods During Visual and Olfactory Trials

**Figure 5:**
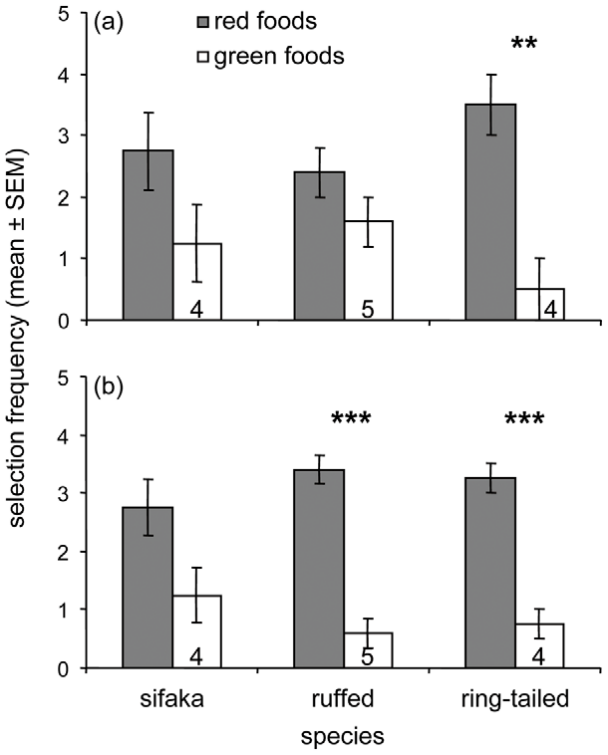
Food-quality preferences during visual and olfactory trials. Food choices of three strepsirrhine species are depicted (for individuals that showed relatively strong food preferences during baseline trials) when (A) only visual or (B) only olfactory cues were available. Presented are the frequencies (mean ± standard error of mean) with which the animals selected red foods (i.e., young leaves or ripe fruits; shaded bars) versus green foods (i.e., mature leaves or unripe fruit; open bars) when only one sensory cue was available. Numbers at the bottom of the open bars represent the number of individuals used in the analysis (G-test: * *P*<0.05, *** *P*<0.001).

The animals that showed relatively strong preferences for red foods during baseline trials, when both visual and olfactory cues were simultaneously available (Figure 4B), generally continued to preferentially select red over green foods when only one sensory cue was available; however, there were some interesting species differences (Figure 5). Notably, when only visual cues were available, only the ring-tailed lemurs maintained a significant preference for red over green foods (*n* = 4; *G*_1_ = 10.12, *P*<0.01), as similar trends failed to reach statistical significance for either the sifakas (*n* = 4; *G*_1_ = 2.31, *P = *0.13) or the ruffed lemurs (*n* = 5; *G*_1_ = 0.81, *P = *0.37; Figure 5A). When only olfactory cues were available, both the ruffed lemurs (*G*_1_ = 10.82, *P*<0.001) and the ring-tailed lemurs (*G*_1_ = 6.74, *P*<0.01) maintained significant preferences for red over green foods, whereas a similar trend failed to reach statistical significance for the sifakas (*G*_1_ = 2.31; Figure 5B, *P* *= *0.12). Thus, sifakas required the simultaneous availability of both visual and olfactory information to reliably identify their preferred food, ruffed lemurs required olfactory cues alone to accurately identify their preferred food, and ring-tailed lemurs could use either sense independently to reliably identify their preferred food. The detailed analyses of behavior for the ruffed and ring-tailed lemurs only revealed additional species differences, in that the generalist ring-tailed lemurs potentially required more time to accurately assess the available information (see Text S4).

Lastly, to ensure that our conservative selection of lemurs with relatively strong red-food preferences in baseline trials did not bias the results for the visual and olfactory trials, we repeated the analyses described above using either all of the study subjects or only those that showed statistically significant preferences for red foods (see Table S2, Text S5). The results were generally consistent across all three sets of analyses.

### Sensory Reliances During Multi-Sensory Trials

**Figure 6:**
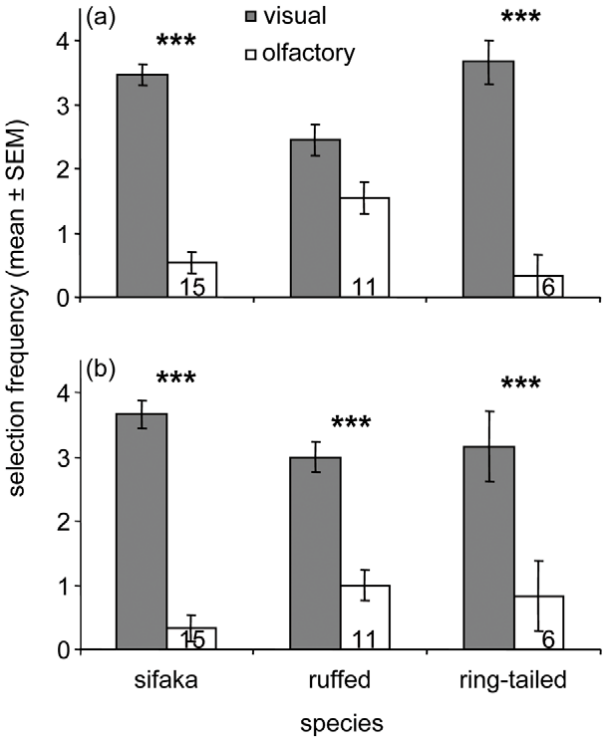
Sensory reliances during multi-sensory trials. Sensory use by three strepsirrhine species (including all subjects tested) when investigating comparable (A) red foods or (B) green foods during multi-sensory trials. Presented are the frequencies (mean ± standard error of mean) with which the animals relied on visual cues (shaded bars) versus olfactory cues (open bars) to select a given food type. Numbers at the bottom of the open bars represent the number of individuals used in the analysis (G-test: *** p<0.001).

When food quality or color was held constant, but both sensory cues were independently available, all members of the three primate species studied generally showed significant biases for relying on their visual sense over their olfactory sense in making their food selection. Both the sifakas (*n* = 15) and the ring-tailed lemurs (*n* = 6) showed consistent visual biases, whether selecting red foods (sifakas: *G*_1_ = 36.06, *P*<0.001; ring-tailed lemurs: *G*_1_ = 19.50, *P*<0.001; Figure 6A) or green foods (sifakas: *G*_1_ = 52.54, *P*<0.001; ring-tailed lemurs: *G*_1_ = 8.71, *P*<0.01; Figure 6B). For ruffed lemurs (*n* = 11), however, the reliance on vision was less pronounced, achieving statistical significance for green foods only (red fruits: *G*_1_ = 2.29, *P = *0.13, Figure 6A; green fruits: *G*_1_ = 11.51, *P*<0.001, Figure 6B). Thus, for red foods, ruffed lemurs were just as likely to rely on vision as on olfaction (Figure 2D–F).

When we compared the strength of sensory reliance among the three species, we found no species difference in visual reliance (red food items: *G*_2_ = 2.77, *P* = 0.25; green food items: *G*_2_ = 0.91, *P* = 0.63); however, there were significant species differences in olfactory reliance (red food items: *G*_2_ = 9.52, *P*<0.01; green food items: *G*_2_ = 6.40, *P*<0.05). Specifically, when red foods were available, ruffed lemurs relied on olfactory cues more than did sifakas (*G*_1_ = 6.70, *P*<0.05) or ring-tailed lemurs (*G*_1_ = 6.18, *P*<0.05); when green foods were available, ruffed lemurs again relied on olfactory cues more than did sifakas (*G*_1_ = 5.93, *P*<0.05). In our more detailed analysis of investigatory behavior by ruffed and ring-tailed lemurs only, we also found significant species differences in sensory reliance when animals selected red foods, but not when they selected green foods (Figure S2).

## Discussion

Animals rely on a suite of sensory cues to search for, identify, and acquire appropriate foods, and when food signals are multimodal, their senses can function synergistically to improve foraging efficiency [61], [62]. Indeed, when selecting fruits or leaves (the varying quality of which was reflected via multimodal signals), the foraging sifakas, ruffed lemurs, and ring-tailed lemurs in our study used their combined visual and olfactory senses to reliably select purportedly high-quality over low-quality food items. Thus, vision and olfaction can function synergistically to optimize the foraging decisions of lemurs. When we manipulated food quality and the sensory cues available to these foraging lemurs, we found that, generally, our study species could use visual or olfactory cues to identify the higher-quality red foods, but they tended to rely more on visual cues than on olfactory cues. Notably, however, consistent with recent genetic data representing a broad range of mammals [25], we found significant species differences in sensory reliance that might relate to differences in the animals' feeding ecology.

As predicted for folivores, the sifakas relied relatively more on vision than olfaction when investigating various foods; nevertheless, they apparently required the simultaneous availability of both visual and olfactory information to reliably identify their preferred foods. By comparison, the frugivorous ruffed lemurs accurately identified their preferred foods using olfactory cues alone. More specifically, when the more numerous volatiles associated with red, ripe fruits (relative to green, unripe fruits) were available, ruffed lemurs were just as likely to rely on olfaction as on vision; however, when the volatile richness associated with ‘ripeness’ was either absent or diminished, these frugivores, like the other strepsirrhines in this study, relied more on vision than olfaction. Lastly, only the generalist ring-tailed lemurs could use either sense independently to reliably identify their preferred foods, but they spent more time looking at foods prior to making their selection than did the ruffed lemurs. While the latter finding may be suggestive of a cost in efficiency to the generalist [59], it remains to be seen if ring-tailed lemurs indeed sacrifice speed over accuracy when making their food choices.

Our results lend support to a growing body of work showing that primates rely heavily on visual cues while selecting food (e.g., [63], [64]); however, it is also clear from our results that both types of sensory cues relay information about food quality, and that some animals can rely relatively more on odor cues. The integration of multimodal sensory information during foraging is prevalent throughout the animal kingdom (e.g., bees [62]; fish [65]; birds [66]). Among strepsirrhines, Piep and colleagues [63] showed that nocturnal mouse lemurs detect more prey if they are provided with a combination of acoustic, visual, and olfactory cues than if only one or two of these modalities are available in isolation. Future field studies, similar to work conducted in New World monkeys [64], [67], could help relate our experimental findings to larger populations of animals foraging in the natural environment and could specifically explore the additive benefits of vision and olfaction in diurnal strepsirrhines.

Compared to the role of vision (particularly color vision), the role of olfaction in primate foraging has received little attention. Studies on olfactory reliance during primate foraging have focused largely on the ability of insectivorous primates to locate concealed food [68]–[70]; only a handful of researchers, primarily working on haplorhines (e.g., [71], [72]), have examined the role of olfaction during foraging by generalist, folivorous, or frugivorous primates. In studies of wild, frugivorous New World monkeys, researchers showed that the animals used olfaction to differentiate between ripe and unripe fruits when visual cues were cryptic (spider monkeys [64]; white-faced capuchins [67]). Similarly, we found that olfaction played an important role in food-quality discrimination for strepsirrhines, particularly for the frugivores. Although we found that olfactory cues may be more informative to the frugivores than the folivores, comparable studies of sensory reliance in haplorhines with varying feeding ecologies are lacking.

Additionally, little is known about the influence of color-vision status on the olfactory reliance of foraging primates. When foraging for visually cryptic fruit, dichromatic capuchins relied more on olfaction than did trichromatic capuchins [67], whereas color-vision phenotype did not play a role in the use of olfactory cues for fruit-eating spider monkeys [64]. In our study, we detected no reliable pattern in the olfactory reliance of dichromats and trichromats, but we did not design our study to test this question and our small sample size precludes generalization. Given recent controversy (e.g., [73], [74]), over the long-held view that olfactory capabilities have decreased specifically with the acquisition of full color vision, it would be interesting to see if dichromatic and trichromatic strepsirrhines, or other primates, e.g., [64] rely differentially on olfactory cues while foraging.

Consistent with other chemical studies [43], [49], our GCMS analyses revealed that red, ripe fruits emitted almost twice as many volatiles as did green, unripe fruits. By contrast, the volatile bouquets of red, young leaves contained only half the number of volatiles as did those of green, mature leaves. The difference in volatile number or ‘richness’ (for further definitions of chemical diversity indices see [75]) associated with different food types may influence cue salience and partially explain species differences in sensory reliance. For instance, the increased chemical richness of ripe fruits may have facilitated their olfactory detection by the frugivorous ruffed lemurs, whereas the potentially decreased chemical salience of young leaves may have encouraged visual reliance in the folivorous sifakas. Interestingly, field researchers have observed a closely related sifaka species, *Propithecus diadema*, using olfactory behavior to locate the visually inconspicuous, but strongly scented inflorescences of subterranean plants [76]. Like fruits, flowers produce aromatic, mature stages (e.g., [77]). Thus, assuming possession of adequate olfactory capabilities, even a visually oriented species might rely on olfaction when the odoriferous cues are sufficiently salient.

Odor cue salience may not necessarily relate to volatile richness, however, in that a single, salient compound may be sufficient to influence food detection (akin to a ‘pheromone concept’ in insect scent signaling [78]). Indeed, the two leaf stages in our study were discernible to the human nose, with young leaves producing a more aromatic fragrance than old leaves despite their sparser volatile profile. An alternate scenario, however, may be required to account for detecting gradual changes associated with the ripening process: It is possible that foraging lemurs respond to multiple volatile compounds in food that interact in different combinations or proportions (consistent with an ‘odor mosaic’ [79] or ‘chemical image’ concept in mammalian scent signaling [80]).

As in other mammals [81], olfactory communication is prominent among strepsirrhines [68], with animals leaving chemically informative messages in various bodily excretions [82] and secretions [83]. Our study species are thus equipped to handle complex olfactory signals. To illustrate the complexity of chemosignaling in this clade, male and female ring-tailed lemurs can use genital scent marks alone (containing >300 volatile compounds) to advertise a wide range of information about themselves (e.g., species, sex, individual identity, genetic quality, and relatedness: [75], [84]–[87]), all of which is decipherable by conspecifics [86], [88], [89]. Given their reliance on olfaction to detect the wide range of information contained in scent marks, it is somewhat surprising that our study species did not show stronger olfactory reliance while foraging. Perhaps relative reliance on the different senses varies by behavioral context (e.g., [90]), with olfaction playing a relatively greater role in social behavior and vision playing a relatively greater role in foraging behavior.

Another factor potentially contributing to variation in sensory reliance between species is that, depending on habitat, the two senses may differentially contribute to facilitating different phases of food selection. In certain environments, for instance, vision (including trichromacy) potentially provides longer-range cues influencing choice of food patch [23] and olfaction (potentially combined with tactile investigation) may provide shorter-range cues influencing choice of specific food items within the patch [91]. Piep and colleagues [63] proposed a similar scenario for how mouse lemurs might use multiple senses during various phases of foraging for arthropod prey. Notably, they might rely on a combination of acoustic cues to detect prey, on visual cues to key in on the prey's location, and on close-range olfactory cues to catch the prey. Although there is little evidence of primates using olfactory cues for long-range foraging [91], there is anecdotal evidence that primates frequently smell fruits at a close range prior to selecting and eating individual fruits [92], [93].

It is clear that further research is necessary to determine the role of chemical cue specificity in animal response elicitation [25]. A new study of OR capabilities and interspecific variation in OR ortholog responsiveness provides one means for examining species-specific proclivities and shows that primates tend to be conserved in their ligand selectivity, differing instead in their response potency [94]. Such an approach could help resolve the extent to which different species respond to specific volatiles associated with differences in food quality. Additionally, generalists, such as the ring-tailed lemur, could be tested on their sensory reliance in detecting a broader range of foods, specifically to address if they rely more on olfaction to detect preferred fruit, but more on vision to detect preferred leaves.

In closing, Endler [95] suggested that sensory systems co-evolve with signaling behavior and habitat occupation, and that such a co-evolution may have led to the wide diversity of sensory systems that exist in the animal kingdom. As evidence of this relationship, the varied reliance on olfaction and echolocation in different species of fruit bats corresponds to the growth patterns of their preferred fruits [96]. Likewise, hummingbirds and passerines have drastically different spectral sensitivities that relate to the different pigmentation patterns of their preferred flowers [97]. Additional links between activity patterns and sensory reliance during foraging are illustrated by olfactory versus visual reliance in nocturnal and diurnal hawkmoths, respectively [98]. Many diurnal birds of prey similarly use UV vision to locate prey ranges that are delineated by UV-visible scent marks [99], whereas at least one species of nocturnal owl lacking UV vision relies more on auditory cues when hunting [100]. Lastly, in studies on sexual selection, researchers have shown a link between foraging for carotenoid-rich foods and color-based mate preferences (e.g., guppies [101], three-spined sticklebacks [102]). Our foraging results for lemurs occupying different ecological niches fit well within this framework and further illustrate how the comparative study of behavior, particularly when informed by comparative molecular (genetic [25], receptor response [94]) studies, can help elucidate the evolution of sensory systems.

## Supporting Information

Figure S1 **Representative chromatograms of the most highly volatile chemicals detected from the multi-sensory panel of the test apparatus, using an electronic sensor.** Shown are the volatiles detected from (A) an empty, open drawer, (B) a green leaf inside a closed, but clear and solid ‘visual’ drawer, and (C) a green leaf inside a closed, but opaque and pierced ‘olfactory’ drawer. Each peak corresponds to a specific volatile compound and has an associated retention time (s) on the x-axis that is specific for the column and analysis temperature. The area under the peak is the compound concentration expressed in counts (cts) on the y-axis.
(DOCX)

Figure S2 **Investigatory behavior during multi-sensory trials.** Duration of behavior by two strepsirrhine species when investigating comparable (A) red foods and (B) green foods during multi-sensory trials. Presented is the time spent (mean ± standard error of mean) looking at (shaded bars) and sniffing (open bars) the food items. Numbers at the bottom of the open bars represent the number of individuals used in the analysis (*t*-test: * p<0.05).
(DOCX)

Table S1 **Experimental study subjects and their food/sensory preferences.** Bolded subjects represent those individuals that showed a relatively strong preference (*P*<0.05 or *P*<0.10 by *G*-test) for red food items during baseline trials. Only these bolded subjects were retained for analyses of performance during visual and olfactory trials, as these latter tests evaluate sensory reliance in animals selecting their preferred high-quality foods. Because multi-sensory trials held food quality constant, all subjects were included in the analyses of these trials.
(DOCX)

Table S2 **Comparison of** ***G*** **-test results for different categories of subjects during visual and olfactory trials.** The subject categories included the following: (1) all of the subjects, (2) only those subjects that showed relatively strong preferences for red foods, and (3) only those subjects that showed significant preferences for red foods. Bolded *G*-tests are significant at *P*<0.05.
(DOCX)

Text S1 **Subject visual status and its effect on performance.** The effect of opsin gene polymorphism (i.e., dichromacy versus trichromacy) on the performance of strepsirrhine primates.
(DOCX)

Text S2 **Volatile compounds in test food items.** Methods for extracting and characterizing the volatile compounds in test food items, and for comparing the volatile compositions of red versus greed food items.
(DOCX)

Text S3 **Apparatus validation with electronic sensor technology.** Use of zNose® technology to validate the functionality of the sensory panels.
(DOCX)

Text S4 **Analyses of the duration of investigatory behavior.** Detailed analyses of looking and sniffing behavior of ruffed lemurs and ring-tailed lemurs.
(DOCX)

Text S5 **Analyses of visual and olfactory trials for different categories of subjects.** Analyses of the performance of (1) all subjects, (2) subjects with relatively strong preferences for red foods, and (3) subjects with significant preferences for red foods.
(DOCX)

Video S1 **Performance by a male sifaka.** A strepsirrhine primate is seen selecting a higher-quality red leaf over a lower-quality green leaf during a baseline trial in which both visual and olfactory cues about the food items were available.
(WMV)
