# Supplementary figures and images for: Sight or Scent: Lemur Sensory Reliance in Detecting Food Quality Varies with Feeding Ecology
Source: PLoS One. 2012 Aug 3;7(8):e41558. doi: 10.1371/journal.pone.0041558 (PMC3411707; doi:10.1371/journal.pone.0041558)

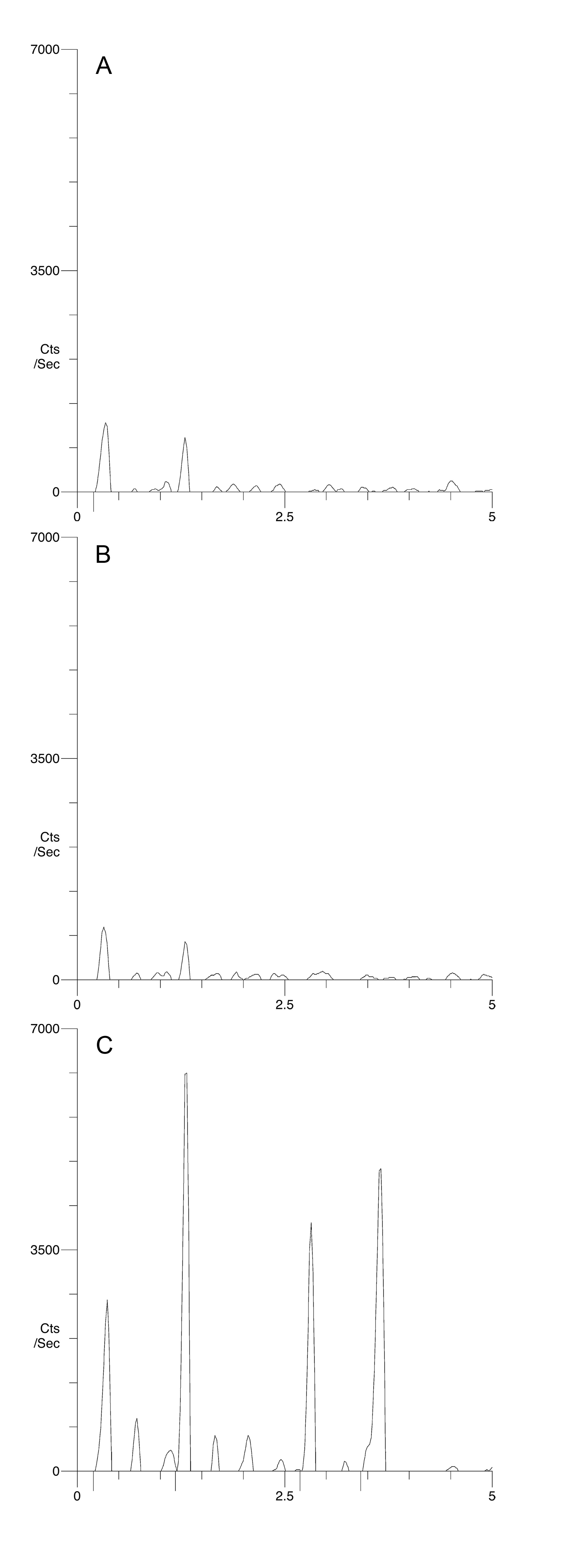

Supplement: Figure S1 — Representative chromatograms of the most highly volatile chemicals detected from the multi-sensory panel of the test apparatus, using an electronic sensor. Shown are the volatiles detected from (A) an empty, open drawer, (B) a green leaf inside a closed, but clear and solid ‘visual’ drawer, and (C) a green leaf inside a closed, but opaque and pierced ‘olfactory’ drawer. Each peak corresponds to a specific volatile compound and has an associated retention time (s) on the x-axis that is specific for the column and analysis temperature. The area under the peak is the compound concentration expressed in counts (cts) on the y-axis. (DOCX) [file pone.0041558.s001.docx]

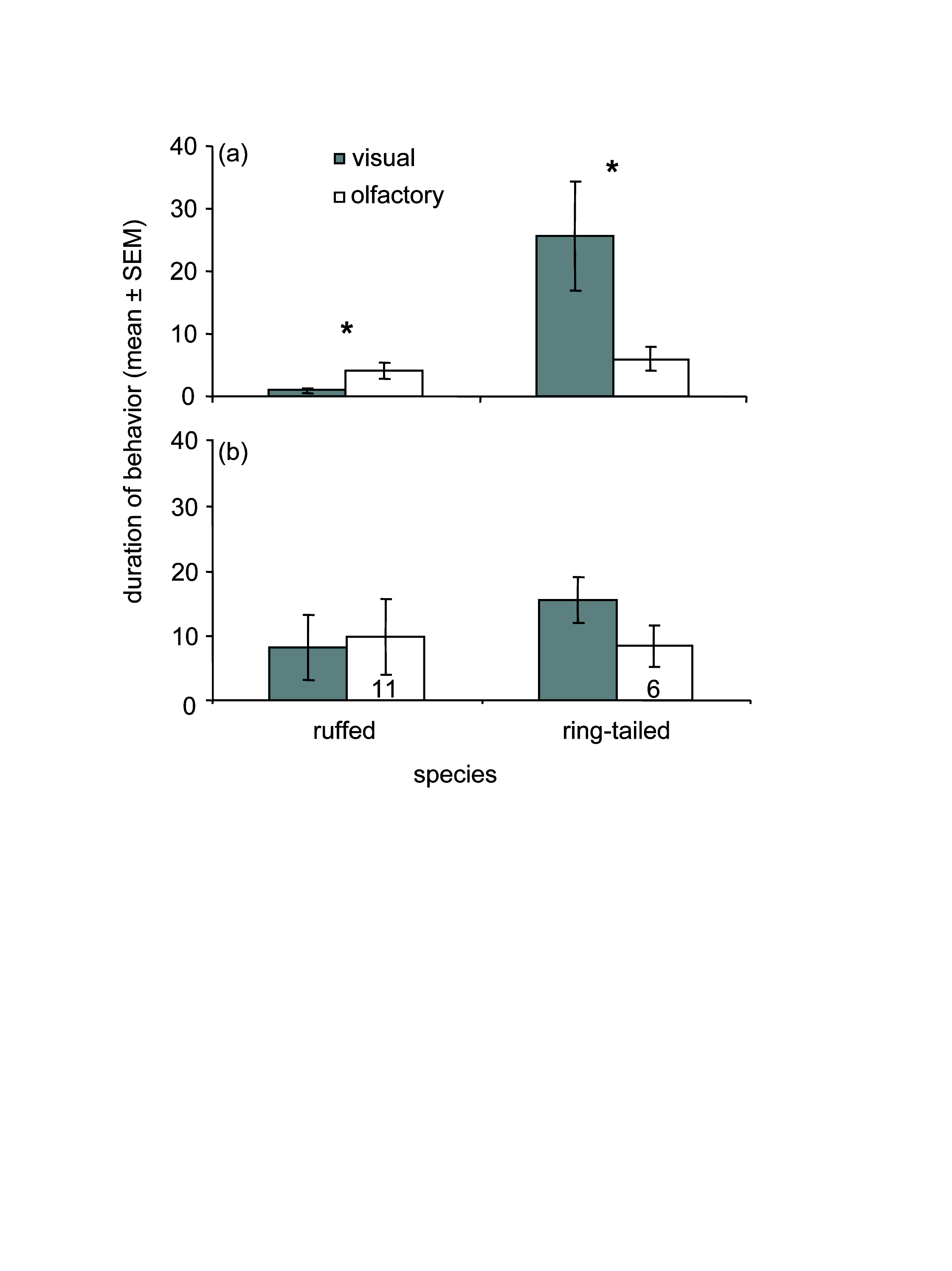

Supplement: Figure S2 — Investigatory behavior during multi-sensory trials. Duration of behavior by two strepsirrhine species when investigating comparable (A) red foods and (B) green foods during multi-sensory trials. Presented is the time spent (mean ± standard error of mean) looking at (shaded bars) and sniffing (open bars) the food items. Numbers at the bottom of the open bars represent the number of individuals used in the analysis (t-test: * p<0.05). (DOCX) [file pone.0041558.s002.docx]
