# Supplementary material for: Sight or Scent: Lemur Sensory Reliance in Detecting Food Quality Varies with Feeding Ecology
Source: PLoS One. 2012 Aug 3;7(8):e41558. doi: 10.1371/journal.pone.0041558 (PMC3411707; doi:10.1371/journal.pone.0041558)
Supplement: Table S1 — Experimental study subjects and their food/sensory preferences. Bolded subjects represent those individuals that showed a relatively strong preference (P<0.05 or P<0.10 by G-test) for red food items during baseline trials. Only these bolded subjects were retained for analyses of performance during visual and olfactory trials, as these latter tests evaluate sensory reliance in animals selecting their preferred high-quality foods. Because multi-sensory trials held food quality constant, all subjects were included in the analyses of these trials. (DOCX) [file pone.0041558.s003.docx]

| **Subjects (DLC #) by subspecies** | **Sex** | **Age (yr) at testing** | **Color vision** | **Percentage of trials in which red was selected** | | | **Percentage of trials in which visual cues were selected** | |
| --- | --- | --- | --- | --- | --- | --- | --- | --- |
|  |  |  |  | **Baseline trials** | **Visual trials** | **Olfactory trials** | **Multi-sensory red trials** | **Multi-sensory green trials** |
| *Propithecus verreauxi coquereli* (*n* = 15) | | | |  |  |  |  |  |
| 6825 | f | 2 | trichromatic | 0.3 | 0.75 | 0.75 | 1 | 1 |
| **6538** | f | 13 | dichromatic | **0.7** | **0.25** | **0.75** | 0.75 | 1 |
| 6727 | f | 8 | dichromatic | 0.45 | 0.5 | 0.5 | 1 | 1 |
| 6743 | f | 7 | dichromatic | 0.5 | 0.75 | 0.5 | 0.75 | 1 |
| 6770 | f | 6 | dichromatic | 0.45 | 0.25 | 0 | 1 | 1 |
| 6828 | f | 2 | dichromatic | 0.65 | 0.5 | 0.75 | 0.5 | 0.75 |
| **6850** | f | 1 | dichromatic | **0.7** | **0.75** | **0.5** | 1 | 1 |
| 6518 | m | 13 | dichromatic | 0.6 | 0.5 | 0.25 | 0.75 | 1 |
| 6583 | m | 12 | dichromatic | 0.55 | 0.75 | 0.5 | 0.75 | 1 |
| 6650 | m | 11 | dichromatic | 0.55 | 0.75 | 0.25 | 1 | 1 |
| **6747** | m | 7 | dichromatic | **1** | **1** | **1** | 1 | 0.75 |
| **6797** | m | 4 | dichromatic | **0.85** | **0.75** | **0.5** | 0.75 | 0.25 |
| 5944 | m | 3 | dichromatic* | 0.35 | 0.5 | 0.25 | 0.75 | 1 |
| 6823 | m | 2 | dichromatic | 0.35 | 0.75 | 0.25 | 1 | 1 |
| 6827 | m | 2 | dichromatic* | 0.45 | 0.25 | 0.75 | 1 | 1 |
| *Varecia variegata rubra* (*n* = 9) | | | |  |  |  |  |  |
| 6377 | f | 16 | trichromatic | 0.65 | 1 | 0.5 | 0.5 | 0.5 |
| 6424 | f | 15 | trichromatic | 0.55 | 0.75 | 0.25 | 0.75 | 0.75 |
| **6633** | f | 11 | trichromatic | **0.8** | **0.75** | **0.75** | 0.5 | 0.75 |
| 6838 | f | 2 | trichromatic | 0.65 | 0.5 | 0.5 | 0.5 | 0.5 |
| **6311** | f | 17 | dichromatic | **0.7** | **0.5** | **0.75** | 0.5 | 0.75 |
| 6802 | f | 4 | dichromatic | 0.3 | 0.75 | 0 | 0.5 | 0.75 |
| **6839** | f | 2 | dichromatic | **0.7** | **0.75** | **1** | 0.25 | 0.5 |
| 6240 | m | 18 | dichromatic* | 0.55 | 0.25 | 0.5 | 0.75 | 1 |
| 6684 | m | 10 | dichromatic* | 0.6 | 0.5 | 0.25 | 0.75 | 1 |
| *Varecia variegata variegata* (*n* = 2) | | | |  |  |  |  |  |
| **2560** | f | 30 | dichromatic | **0.75** | **0.25** | **1** | 0.75 | 1 |
| **5604** | m | 25 | dichromatic | **0.75** | **0.75** | **0.75** | 1 | 0.75 |
| *Lemur catta* (*n* = 6) | | | |  |  |  |  |  |
| **6159** | f | 19 | dichromatic | **0.95** | **1** | **0.75** | 1 | 0.5 |
| 6709 | f | 9 | dichromatic | 0.55 | 1 | 0.75 | 1 | 1 |
| **6857** | f | 1 | dichromatic* | **0.7** | **0.5** | **0.75** | 1 | 1 |
| **6865** | f | 1 | dichromatic* | **0.95** | **1** | **1** | 0.5 | 0.25 |
| **6485** | m | 14 | dichromatic* | **0.85** | **1** | **0.75** | 1 | 1 |
| 6849 | m | 4 | dichromatic* | 0.55 | 0.5 | 1 | 1 | 1 |

***** Animals for which no genetic data were available were presumed to be dichromatic based on their sex (male), their species (*L. catta*), or their mother being dichromatic, as determined by Leonhardt et al. (2009).
