# Supplementary material for: Sight or Scent: Lemur Sensory Reliance in Detecting Food Quality Varies with Feeding Ecology
Source: PLoS One. 2012 Aug 3;7(8):e41558. doi: 10.1371/journal.pone.0041558 (PMC3411707; doi:10.1371/journal.pone.0041558)
Supplement: Table S2 — Comparison of G -test results for different categories of subjects during visual and olfactory trials. The subject categories included the following: (1) all of the subjects, (2) only those subjects that showed relatively strong preferences for red foods, and (3) only those subjects that showed significant preferences for red foods. Bolded G-tests are significant at P<0.05. (DOCX) [file pone.0041558.s004.docx]

| **Trial** | **Species** | **Subjects included** | **Sample size** | **df ^a^** | ***G*-test** | ***P*-value** |
| --- | --- | --- | --- | --- | --- | --- |
| Vision | Sifaka | All | 15 | 1 | 2.42 | 0.120 |
|  |  | Relatively strong preference* | 4 | 1 | 2.31 | 0.129 |
|  |  | Significant preference** | 2 | 1 | 5.06 | **0.024** |
|  | Ruffed | All | 11 | 1 | 2.29 | 0.130 |
|  |  | Relatively strong preference* | 5 | 1 | 0.81 | 0.369 |
|  |  | Significant preference** | 3 | 1 | 0.33 | 0.563 |
|  | Ring-tailed | All | 6 | 1 | 11.64 | **0.001** |
|  |  | Relatively strong preference* | 4 | 1 | 10.12 | **0.001** |
|  |  | Significant preference** | 3 | 1 | 7.92 | **0.005** |
| Olfaction | Sifaka | All | 15 | 1 | 0.07 | 0.793 |
|  |  | Relatively strong preference* | 4 | 1 | 2.31 | 0.129 |
|  |  | Significant preference** | 2 | 1 | 2.09 | 0.148 |
|  | Ruffed | All | 11 | 1 | 0.82 | 0.365 |
|  |  | Relatively strong preference* | 5 | 1 | 10.82 | **0.001** |
|  |  | Significant preference** | 3 | 1 | 5.82 | **0.016** |
|  | Ring-tailed | All | 6 | 1 | 11.64 | **0.001** |
|  |  | Relatively strong preference* | 4 | 1 | 6.74 | **0.009** |
|  |  | Significant preference** | 3 | 1 | 5.82 | **0.016** |

* Subjects showed a relatively strong preference for red foods during baseline trials at a significance level of *P* < 0.05 or *P* < 0.10 or by *G*-test.

*** Subjects showed a significant preference for red foods during baseline trials at a significance level of *P* < 0.05 by *G*-test.

^a^ df refers to degrees of freedom.
