# Supplementary material for: Sight or Scent: Lemur Sensory Reliance in Detecting Food Quality Varies with Feeding Ecology
Source: PLoS One. 2012 Aug 3;7(8):e41558. doi: 10.1371/journal.pone.0041558 (PMC3411707; doi:10.1371/journal.pone.0041558)
Supplement: Text S1 — Subject visual status and its effect on performance. The effect of opsin gene polymorphism (i.e., dichromacy versus trichromacy) on the performance of strepsirrhine primates. (DOCX) [file pone.0041558.s005.docx]

**Subject Visual Status and its Effect on Performance**

Based on prior genotyping [[1](#_ENREF_1)], one of the female sifakas and 4 of the female red-ruffed lemurs that served in the present study had heterozygous alleles at the medium and long-wavelength sensitive X-linked opsin gene, which underlies trichromatic vision. The remaining sifakas, ruffed lemurs, and all of the ring-tailed lemurs were homozygous at this gene, and therefore possessed dichromatic vision. Owing to small sample sizes, we assessed the potential influence of opsin gene polymorphism in ruffed lemurs only.

Under camouflage conditions, trichromacy in strepsirrhines is linked with only modest foraging advantages, namely in detecting red foods against a green, foliar background [[1](#_ENREF_1)]. Because our experimental tasks did not involve camouflage, we did not expect any performance differences between dichromats and trichromats. Indeed, in ruffed lemurs, we found no differences in baseline preferences by visual status, with dichromats (*n* = 7, *G_1_* = 8.34, *P <* 0.001) and trichromats (*n* = 4, *G_1_* = 8.61, *P* < 0.001) showing comparable preferences for red over green food items.

Other results on the effects of visual status were somewhat mixed. For instance, among those five ruffed lemurs that individually showed relatively strong preferences for red food items during baseline trials (i.e., *P* < 0.05 or *P* < 0.10 by *G*-test), only one was trichromatic. Thus, whereas 57% of the dichromatic ruffed lemurs showed relatively strong preferences for red food items, only 25% of the trichromatic ruffed lemurs showed relatively strong preferences for red food items. During visual trials, however, the trichromats preferentially selected red over green food items (*n* = 4, *G_1_* = 4.19, *P <* 0.05), whereas the dichromats did not (*n* = 7, *G_1_* = 0.14, *P >* 0.70). As no consistent pattern differentiated the performance of these two groups, we collapsed dichromats and trichromats in the subsequent analyses.

**References**

1. Leonhardt SD, Tung J, Camden JB, Leal M, Drea CM (2009) Seeing red: behavioral evidence of trichromatic color vision in strepsirrhine primates. Behav Ecol 20: 1-12.
