# Supplementary material for: Sight or Scent: Lemur Sensory Reliance in Detecting Food Quality Varies with Feeding Ecology
Source: PLoS One. 2012 Aug 3;7(8):e41558. doi: 10.1371/journal.pone.0041558 (PMC3411707; doi:10.1371/journal.pone.0041558)
Supplement: Text S2 — Volatile compounds in test food items. Methods for extracting and characterizing the volatile compounds in test food items, and for comparing the volatile compositions of red versus greed food items. (DOCX) [file pone.0041558.s006.docx]

**Volatile Compounds in Test Food Items**

**Extraction and characterization of the volatile compounds in test food items**

We harvested 400 mg of fresh *Photinia spp.* leaves and 4 g of fresh *S. lycopersicum* fruits from organic gardens to preclude contamination with pesticides. We transferred the leaf strips (torn to 5 mm in length) or diced tomatoes into airtight, 50-ml vials. The leaves and tomatoes filled the vials to approximately equal volumes. To collect the volatiles emitted, we used a SPME method with an 85µm Car-PDMS fiber (Supelco, Sigma-Aldrich, Bellefonte, PA). We incubated samples above a water bath at 50°C. We manually injected the volatiles collected from each sample, into a GC (Shimadzu QP2010, Shimadzu, Kyoto, Japan) by introducing the SPME fiber directly into the GC-inlet.

The GC was equipped with a Restek SHR5XB column (30 m x 0.25 mm x 0.25 µm, Shimadzu) and coupled to a high-resolution mass selective detector (Shimadzu QP2010). Volatiles were desorbed at an injector temperature of 280°C. Helium was used as the carrier gas at a flow rate of 1 ml/min. We set the ion source temperature at 250°C and scanned masses from 80 to 525 amu/Daltons in 0.5 sec. The oven temperature was programmed from 50°C to 180°C at 10°C/min (hold time: 2 min) and then to 320°C at 20°C/min (hold time: 3 min). The electron impact mass spectra were recorded at 250°C. Runs lasted 25 min.

We ran 9-10 samples per plant species, with approximately equal representation of each developmental stage. To ensure that compounds did not accumulate in the column, we performed a blank run after every 5 runs. We used the Windows version of the GCMS solution software package (Shimadzu QP2010, Shimadzu, Kyoto, Japan) for data acquisition. The volatile compounds found in the headspace of leaves and fruits were characterized by their mass spectra, diagnostic ions, and retention times. Peaks with identical mass spectra and retention times were regarded as the same substance.

**Analysis of the volatile chemicals in red and green test food items**

After excluding all chromatogram peaks that individually accounted for less than 0.1% of the total peak area, we examined the differences in the total number of compounds found in red and green *Photinia* leaves and *S. lycopersicum* fruits. To examine the chemical variation between red and green foods, we used R-software [[1](#_ENREF_2)] to perform two principle component analyses (PCA), one for the leaves and another for the tomatoes. The analyses were based on a total of 32 compounds, quantified as relative proportions by dividing the peak area of each compound by the total area of all the peaks in the chromatogram. The results for this analysis are presented in the main text (Figure 3).

**References**

1. R Development Core Team (2007) R: A language and environment for statistical computing. R Foundation for Statistical Computing.
