# Supplementary material for: Sight or Scent: Lemur Sensory Reliance in Detecting Food Quality Varies with Feeding Ecology
Source: PLoS One. 2012 Aug 3;7(8):e41558. doi: 10.1371/journal.pone.0041558 (PMC3411707; doi:10.1371/journal.pone.0041558)
Supplement: Text S3 — Apparatus validation with electronic sensor technology. Use of zNose® technology to validate the functionality of the sensory panels. (DOCX) [file pone.0041558.s007.docx]

**Apparatus Validation with Electronic Sensor Technology**

The zNose® is a portable device that is based on ultra-fast gas chromatography (GC) and performs analytical measurements of volatile organic vapors and odors, *in situ*, in near real time. It produces high-resolution 2D olfactory images of aroma chemistry, with part-per-trillion sensitivity, and provides the purest image of odorants, as no components are lost through storage or solvent extraction procedures.

Using the multi-sensory panel, we placed similar test food items in each drawer. For each side separately, we captured any volatiles emitted by aspirating the vapor around the front of the panel and its associated drawer. We also aspirated room air as a control. The volatiles were desorbed and passed, via helium carrier gas, through the GC column for separation. After passing through the column, the compounds hit a sensory acoustic wave detector where they change the detector’s inert frequency. The frequency shift caused by each analyte is characteristic of the amount of material hitting the detector, thereby allowing quantification.

The results of our validation procedures are illustrated by representative chromatograms in Figure S1.
