# Supplementary material for: Sight or Scent: Lemur Sensory Reliance in Detecting Food Quality Varies with Feeding Ecology
Source: PLoS One. 2012 Aug 3;7(8):e41558. doi: 10.1371/journal.pone.0041558 (PMC3411707; doi:10.1371/journal.pone.0041558)
Supplement: Text S4 — Analyses of the duration of investigatory behavior. Detailed analyses of looking and sniffing behavior of ruffed lemurs and ring-tailed lemurs. (DOCX) [file pone.0041558.s008.docx]

**Analyses of the Duration of Investigatory (Looking and Sniffing) Behavior**

In addition to analyzing the number of times the lemurs selected food items or sensory cues, we also examined the time that ruffed lemurs and ring-tailed lemurs spent looking at or sniffing food items (sifakas were not included for logistical reasons). Looking involved a lemur directing its gaze (head and eyes) toward a food item, which was visible through the transparent wall of the foraging apparatus; sniffing involved a lemur inspecting the scent holes above a drawer with its nose, which involved jutting the head forward to direct the nostrils toward the food item. Behavior occurred maximally within 25 cm of the apparatus and, for our purposes, looking and sniffing were considered to be mutually exclusive.

To analyze differences between the ruffed lemurs and ring-tailed lemurs in the duration of looking and sniffing during visual and olfactory trials, we used *t*-tests in which we log-transformed duration data (to meet parametric test assumptions). We found that ring-tailed lemurs spent more time than did ruffed lemurs looking at red fruits during visual trials (*t*_14.73_ = 9.11, *P <* 0.001) and sniffing red fruits during olfactory trials (*t*_12.75_ = 3.42, *P* < 0.01). Thus, although ring-tailed lemurs were equally successful at identifying red foods using either sense, they potentially required more time to accurately assess the available information.

To compare the time the subjects spent looking at versus sniffing food items prior to making a selection during red and green multi-sensory trials, we used two-tailed, paired *t*-tests. We also used *t*-tests to analyze species differences in the time spent looking at versus sniffing food items, which could serve as a proxy for estimating efficiency. We found significant species differences in sensory reliance when animals selected red foods, but not when they selected green foods (Figure S2). Specifically, when presented with red foods, ruffed lemurs generally sniffed the food (*t*_10_ = 3.09, *P* < 0.05), whereas ring-tailed lemurs generally looked at the food (*t*_5_ = -2.86, *P* < 0.05, Figure S2a). These sensory biases were absent in both species when presented with green foods (ruffed: *t*_10_ = 0.16, *P* = 0.88; ring-tailed: *t*_5_ = -1.99, *P* = 0.10, Figure S2b). Ring-tailed lemurs spent significantly more time looking at food items than did ruffed lemurs (red: *t*_8.37_ = 7.92, *P* < 0.001; green: *t*_13.96_ = 3.15, *P* < 0.01), but both species sniffed the food items equally (red: *t*_9.02_ = 0.79, *P* = 0.45; green: *t*_12.44_ = 0.69, *P* = 0.50).

Overall, the ring-tailed lemurs spent more time looking at foods prior to making their selection than did the ruffed lemurs. Although this finding may be suggestive of a cost in efficiency to the generalist, consistent with the speed-accuracy trade-off observed in other species [[54](#_ENREF_54)], it remains to be seen if ring-tailed lemurs indeed sacrifice speed over accuracy when making these choices.
