# Supplementary material for: Sight or Scent: Lemur Sensory Reliance in Detecting Food Quality Varies with Feeding Ecology
Source: PLoS One. 2012 Aug 3;7(8):e41558. doi: 10.1371/journal.pone.0041558 (PMC3411707; doi:10.1371/journal.pone.0041558)
Supplement: Text S5 — Analyses of visual and olfactory trials for different categories of subjects. Analyses of the performance of (1) all subjects, (2) subjects with relatively strong preferences for red foods, and (3) subjects with significant preferences for red foods. (DOCX) [file pone.0041558.s009.docx]

**Analyses of Visual and Olfactory Trials for Different Categories of Subjects**

In the main text, we presented results of *G-*tests limited only to those subjects that showed relatively strong preferences for red foods during the baseline trials (i.e., based on a significance level of *P* < 0.05 or *P* < 0.10 by *G*-test). Table S2 shows a comparison among *G*-tests performed on (1) all of the study subjects, (2) those subjects with relatively strong preferences for red foods, and (3) those subjects with significant preferences for red foods (i.e., based on a significance level of *P* < 0.05 by *G*-test).

The results for these three categories of subjects are generally consistent, with two exceptions. First, when all ruffed lemurs were included in the analysis for olfactory trials, the results showed that they could not use olfactory cues alone to identify red foods (*n* = 11, *G_1_* = 0.82, *P =* 0.365); alternatively, when we examined only ruffed lemurs with relatively strong or significant preferences, the results showed that they could use olfactory cues alone to identify red foods (relatively strong preference: *n* = 5, *G_1_* = 10.82, *P <* 0.01, significant preference: *n =* 3, *G_1_* = 5.82, *P* < 0.05). Similarly, when we included only those sifakas with significant red-food preferences in the analysis for visual trials, the results indicated that they could use olfactory cues alone to identify red foods (*n* = 2, *G_1_* = 5.06, *P <* 0.05); however, when we examined all the sifakas or only those sifakas with relatively strong red-food preferences, the results indicated that they could not identify red food items using visual cues alone (all: *n* = 15, *G_1_* = 2.42, *P =* 0.120, relatively strong preference: *n =* 4, *G_1_* = 2.31, *P* = 0.129). All of the other results were consistent, regardless of which subset of individuals we included in the analysis. Using the subset of lemurs that showed relatively strong preferences for red food items was deemed the most conservative approach, as the results for this subset were always in agreement with the results of at least one other subset (i.e., all of the subjects or only those showing statistically significant preferences for red foods).
